# Perihematomal Edema After Intracerebral Hemorrhage: An Update on Pathogenesis, Risk Factors, and Therapeutic Advances

**DOI:** 10.3389/fimmu.2021.740632

**Published:** 2021-10-19

**Authors:** Yihao Chen, Shengpan Chen, Jianbo Chang, Junji Wei, Ming Feng, Renzhi Wang

**Affiliations:** ^1^ Department of Neurosurgery, Peking Union Medical College Hospital, Peking Union Medical College, Chinese Academy of Medical Sciences, Beijing, China; ^2^ Department of Neurosurgery, Guangdong Provincial People’s Hospital, Guangdong Institute of Neuroscience, Guangdong Academy of Medical Sciences, Guangdong, China

**Keywords:** intracerebral hemorrhage, perihematomal edema, neuroinflammation, pathophysiology, therapies

## Abstract

Intracerebral hemorrhage (ICH) has one of the worst prognoses among patients with stroke. Surgical measures have been adopted to relieve the mass effect of the hematoma, and developing targeted therapy against secondary brain injury (SBI) after ICH is equally essential. Numerous preclinical and clinical studies have demonstrated that perihematomal edema (PHE) is a quantifiable marker of SBI after ICH and is associated with a poor prognosis. Thus, PHE has been considered a promising therapeutic target for ICH. However, the findings derived from existing studies on PHE are disparate and unclear. Therefore, it is necessary to classify, compare, and summarize the existing studies on PHE. In this review, we describe the growth characteristics and relevant underlying mechanism of PHE, analyze the contributions of different risk factors to PHE, present the potential impact of PHE on patient outcomes, and discuss the currently available therapeutic strategies.

## Introduction

The prognosis of patients with hemorrhagic stroke is extremely poor, resulting in long hospital stays and high costs (1). Each year, approximately 2.8 million people die of intracerebral hemorrhage (ICH) worldwide (2), and only 25% of ICH survivors are able to live independently 6 months after ICH onset (3). The functional neurological outcome of ICH is associated with mechanical destruction of nerve fibers and ICH-induced secondary brain injury (SBI).

Perihematomal edema (PHE) manifests when the water content increases in the brain tissue adjacent to the intraparenchymal hematoma. The development of PHE has been considered a quantifiable marker of SBI and is associated with thrombin activation, an inflammatory immune response, blood–brain barrier (BBB) dysfunction, and hemoglobin cytotoxicity after ICH (4–6). PHE also induces a significant mass effect, and rapid growth of PHE may result in severe intracranial hypertension. The International Surgical Trial in Intracerebral Hemorrhage (STICH) I and II showed no clinical benefit of early surgical evacuation of the hematoma in patients with ICH (7, 8); therefore, whether targeted treatment for PHE can provide favorable effects has become of great interest to researchers. Evidence obtained from high-quality preclinical research is required to investigate this issue. A comprehensive understanding of the pathogenesis and natural course of PHE is urgently needed to discover novel therapeutic targets for ICH-induced SBI.

Most research on PHE in patients with ICH has been retrospective. However, it is challenging to obtain good congruity in the timing of head computed tomography (CT) examinations in retrospective studies (9, 10). Previous studies adopted different severity indices and measurements for PHE and used CT scanning more often than head magnetic resonance imaging (MRI) (11, 12). These factors have led to discrepant findings in the exploration of the natural course and prognosis of PHE. In the present review, the PHE literature is assessed to describe the development characteristics of, pathophysiologic mechanisms of, and risk factors for PHE. This review also discusses the impacts of PHE on the clinical outcomes of patients with ICH and the currently available therapeutics for PHE in an effort to provide deeper insights into ICH-induced SBI and provide relevant data for innovative trials.

## Natural Course of PHE

An experimental study of ICH showed that PHE was initiated in the acute phase, peaked at 3 to 4 days, and persisted for 7 days after onset (13). These findings are consistent with the neuropathological changes in experimental animals reported by Enzmann et al. (13), who found significant rupture of perihematomal erythrocytes and a peak perihematomal neuroinflammatory response 4 days after inducement of ICH. Additionally, Sun et al. (14) found that the aquaporin-4 (AQP-4) involved in brain water accumulation peaked at 48 h in a rat model of autologous blood injection.

**Figure 1:**
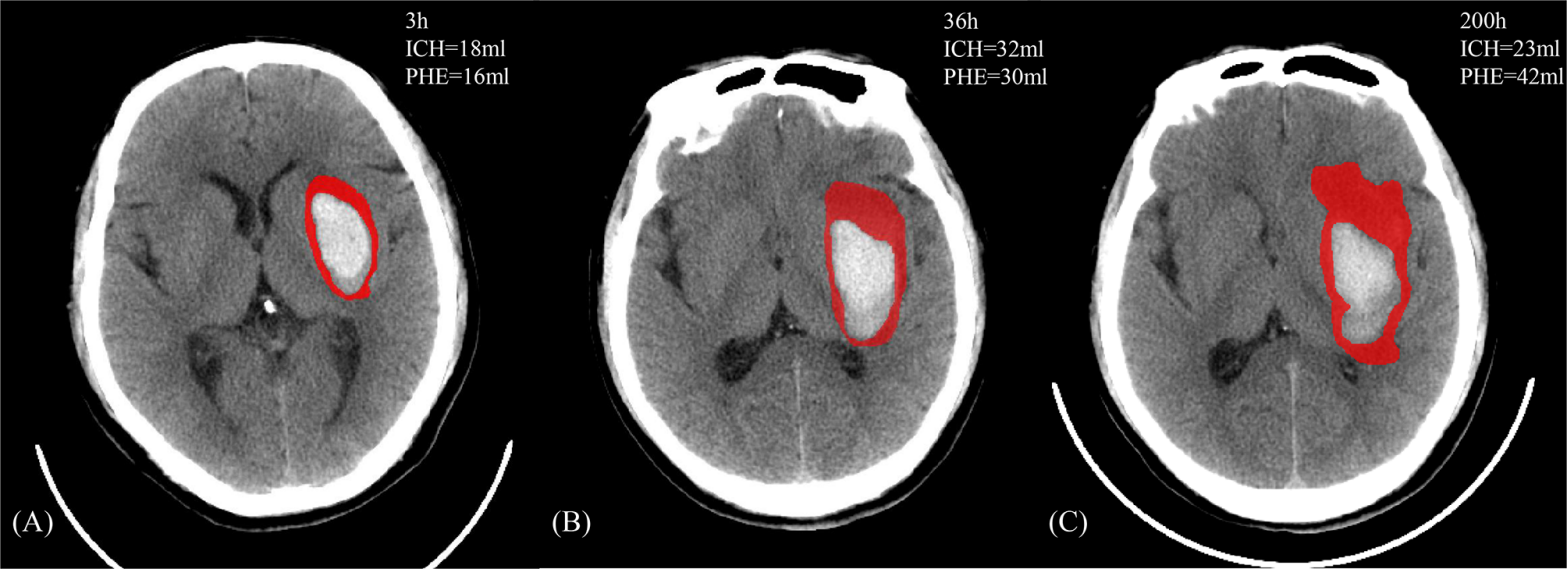
A 47-year-old male who manifested weakness of the right limb and gradually developed mild disturbance of consciousness without clear inducement. There exists a past medical history with hypertension, which the admission blood pressure is 166/106 mmHg and the Glasgow Coma Scale score was 12. The head NCCT revealed left basal ganglia hemorrhage. The patient received the standardized medical management, and the discharge Glasgow Outcome Scale score is 3. **(A**–**C)** The image features of PHE (in red) and ICH against the onset time.

**Figure 2:**
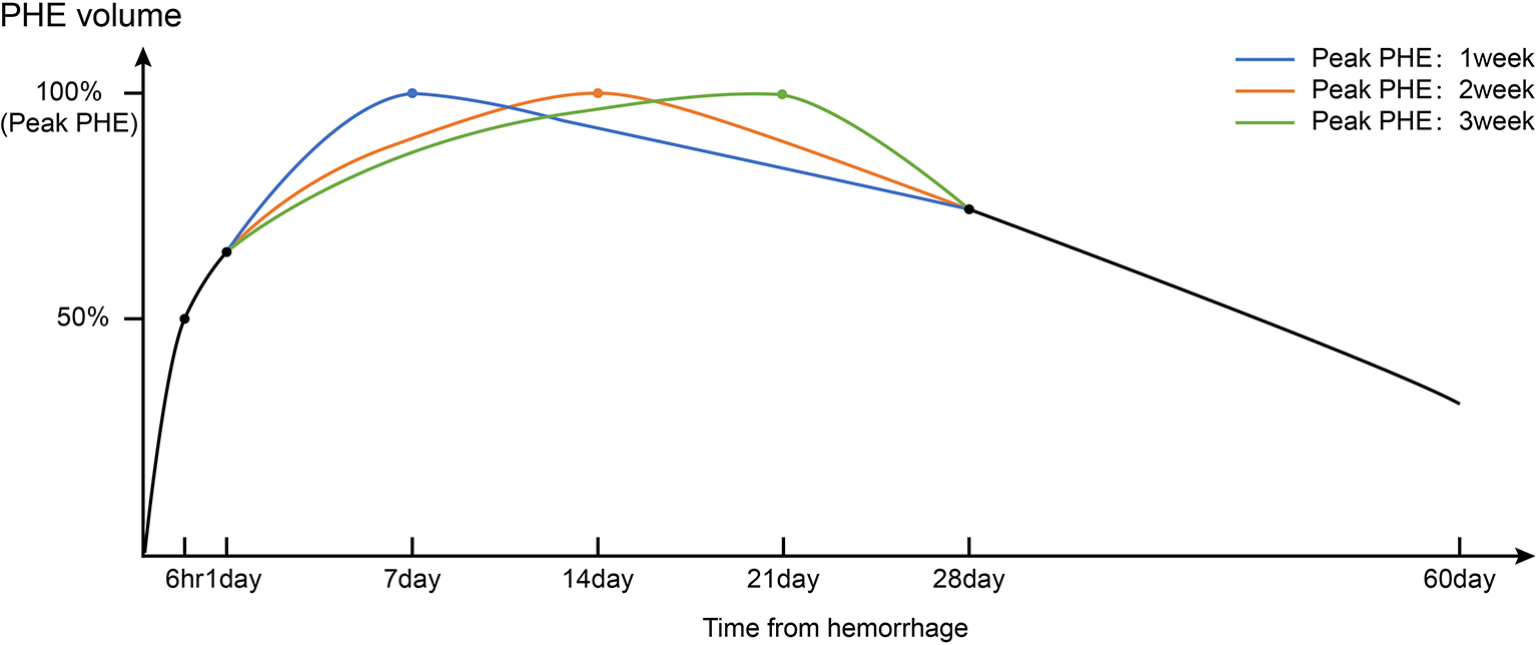
Timeline of PHE volumes after intracerebral hemorrhage based on real-world clinical research of the natural history of PHE. Triphasic patterns of growth of PHE (fast-growing phase: 1–3 days; slow-growing phase and peak period: 1–3 weeks; resorption phase: >3 weeks) were observed.

Because PHE occurs predominantly in white matter, and because a significant discrepancy in the development of white matter exists between humans and animals (especially rodents), PHE growth is expected to be even more prominent in human beings (**Figure 1**) (15). In one human imaging study, all patients with ICH developed PHE within 6 h of symptom onset (12). The ultra-early stage of ICH is commonly regarded as the rapid growth phase of PHE (**Figure 2**). Wu et al. (10) reported that PHE grows rapidly within 24 h after onset and that the edema extension distance (EED) at 24 h accounts for 60% of the peak EED. Other researchers have reported that the time window from symptom onset to 48 or 72 h after symptom onset is the phase of most rapid PHE growth (16, 17). These discrepant findings are partly related to the heterogeneous timing of follow-up CT scans in retrospective studies of patients with PHE as well as the various metrics reflecting the severity of PHE that were adopted among different studies.

The growth rate of PHE gradually decreases after the rapid growth phase (**Figure 2**). Wu et al. (10) reported that the line of best fit between the growth rate of the EED (*y*, cm) and the symptom onset time (*x*, days) can be calculated as follows: *y* = 0.162*x* ^−0.927^ (*R* ^2^ = 0.820). The PHE volume peaks at around 1 to 2 weeks after onset of ICH (17–19). However, growth of PHE in a small number of patients persists up to 3 weeks after onset, which might be associated with a high hematocrit at admission. In contrast, one study showed that an early peak in PHE growth may be associated with rupture of the hematoma into the ventricle (16). This may be clinically relevant because a high hematocrit indicates higher red blood cell (RBC) degradation, which has been identified as an essential factor for promotion of PHE. The abovementioned study showed that in patients with rupture of the hematoma into the ventricle, the deposition of lysed erythrocytes in the brain parenchyma was alleviated by the dilutional effect of the cerebrospinal fluid. Unexpectedly, the initial hematoma volume did not appear to affect the peak time of PHE (16). The peak time points may also differ according to the location of ICH. Sprügel et al. (20) reported that lobar ICH was associated with earlier peak PHE onset and a greater initial PHE volume than deep ICH. This likely occurred because of the irregular shape of the lobar ICH within the relatively loose brain tissue, promoting a higher PHE volume per unit of the hematoma surface area. Therefore, it is clinically important to evaluate the PHE growth patterns when differentiating the common causes of lobar ICH (e.g., cerebral amyloid angiopathy) and deep ICH (e.g., hypertensive ICH). Peng et al. (21) showed that in about 30% of patients, the PHE volume at 2 to 3 weeks after ICH was 3 ml greater than that within 1 week after ICH, and this increase in volume was an independent risk factor for a poor prognosis. However, a clear definition of delayed PHE formation is still lacking.

After peaking, PHE enters a phase in which it slightly decreases in volume (**Figure 2**). One study showed that in patients who did not undergo curative surgery, the PHE volume at 4 weeks was similar to that at 1 week after onset (22). However, Fung et al. (23) found that in patients with a large initial PHE volume, about 60 days was required for the PHE to return to the baseline level regardless of whether the patient had undergone decompression. For patients with ICH who undergo hematoma removal procedures, the natural postoperative course of PHE differs from that before surgery. Horowitz et al. (24) defined postoperative PHE as “pericavity edema” and investigated its time course. They found that pericavity edema grew mainly within the first 2 days postoperatively, after which the growth maintained a steady state. A higher percentage of hematoma removal resulted in slower growth of pericavity edema (24).

## Mechanisms of PHE

### Cytotoxic Edema *vs.* Vasogenic Edema

**Figure 3:**
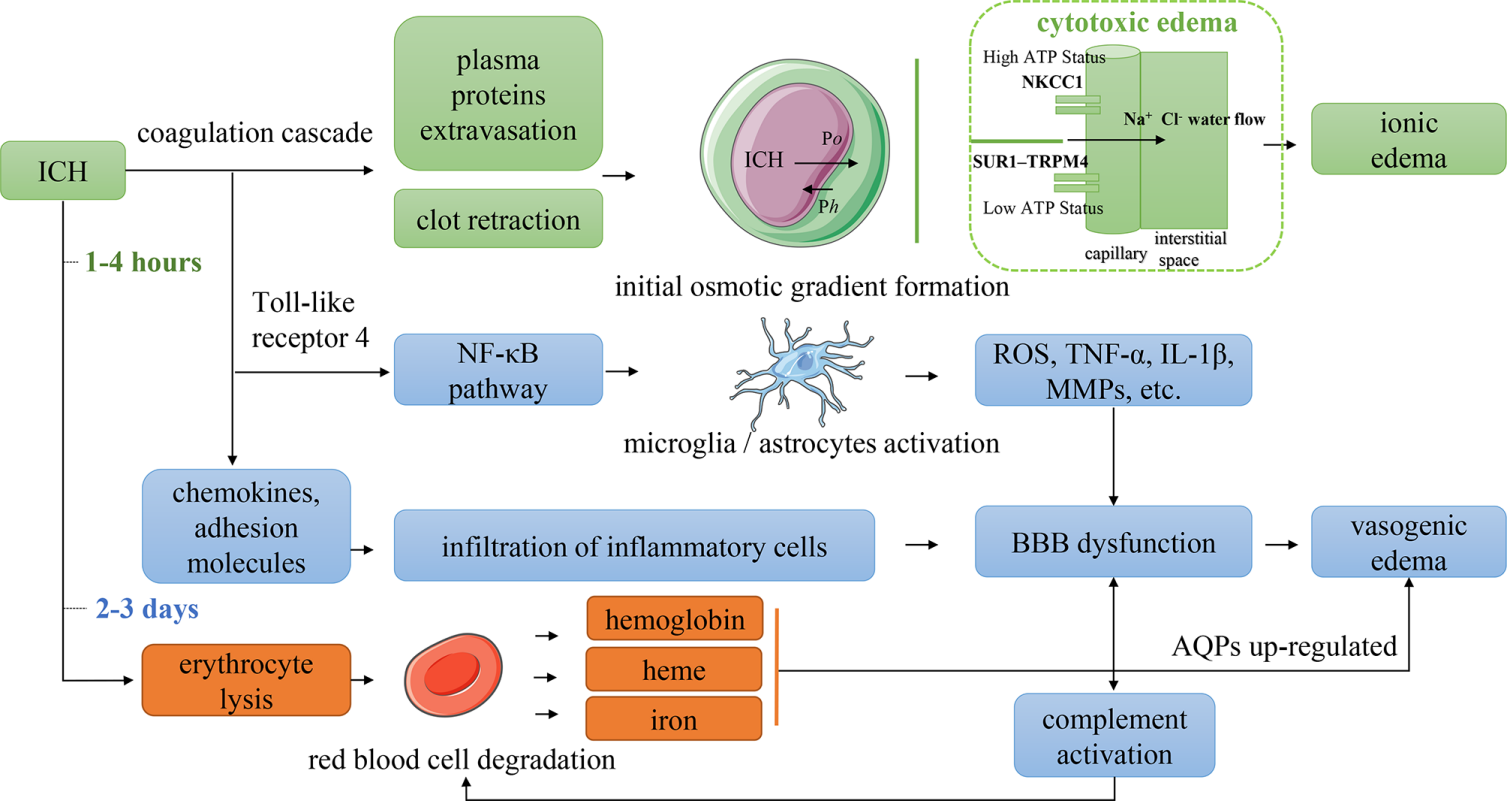
Mechanism of PHE formation. P*o* indicated the difference in oncotic pressure between the capillaries and interstitial tissue. P*h* indicated the difference in hydraulic pressure between the capillaries and interstitial tissue. NKCC1, Na–K–Cl cotransporter 1; SUR1–TRPM4, sulfonylurea receptor 1–transient receptor potential cation channel subfamily M member 4; ROS, reactive oxygen species; TNF-α, tumor necrosis factor-α; IL-1β, interleukin 1β; MMPs, matrix metalloproteinases; BBB, blood–brain barrier; AQPs, aquaporins.

Although the complex mechanism of PHE growth is not yet completely clear, preclinical evidence suggests that different pathophysiologic mechanisms dominate the development of PHE at different stages of ICH (**Figure 3**) (4–6, 25). The essential phenomenon underlying PHE formation is an imbalance of the perivascular fluid interchange based on Starling’s principle (26). Specifically, the formation of an osmotic gradient and the elevation of capillary hydrostatic forces drive the flow of fluids that result in the development of PHE. Starling’s classic formula is *J*_V_ = *K*_H_(*P*_C_ − *P*_I_) − *K*_O_(π_C_ − π_I_), where *J*_V_ is the net transendothelial fluid transfer in brain capillaries; *K*_O_ and *K*_H_ are the filtration coefficients of oncotic conductivity and hydraulic conductivity, respectively; *π*_C_ − *π*_I_ is the difference in oncotic pressure between the capillaries and interstitial tissue; and *P*_C_ – *P*_I_ is the difference in hydraulic pressure between the capillaries and interstitial tissue.

Both cytotoxic edema and vasogenic edema play particularly significant roles in PHE formation. Cytotoxic edema dominates the initial stage of PHE and is a premorbid precursor to extracellular ionic edema resulting from the dysfunction or abnormal activation of ion pumps in endothelial cells and astrocytes. Perihematomal glutamate deposition may contribute to cytotoxic edema (27). The extracellular concentration of glutamate in patients with stroke or traumatic brain injury can be 20 times higher than that in healthy individuals (28, 29). The opening of ion channels increases the movement of water from the extracellular to intracellular space, causing cell swelling and even cell death (30, 31). The essential mechanism of cytotoxic edema is the variation in the brain water distribution, which does not induce true tissue space swelling; however, the transendothelial osmotic gradients derived from cytotoxic edema provide the driving force for ionic edema. Astrocyte swelling is a typical manifestation of cytotoxic edema. When cytotoxic edema occurs, water gains access to the central nervous system through the AQP-4 expressed in astrocytic foot processes (32). Upregulated AQP-4 expression has been identified in patients with ischemic stroke and facilitates ionic edema formation (33). However, how AQP-4 affects ion transcellular transport remains unclear.

Vasogenic edema dominates the second stage of PHE formation, which is characterized by BBB dysfunction caused by a series of neuroinflammatory responses associated with the mechanical destruction of ICH, thrombin activation, and toxic effects of erythrocyte lysis (4–6). Vasogenic edema is a consequence of multifactorial actions. In the immune response associated with neuroinflammation, the disruption of tight junctions between vascular endothelial cells increases vascular permeability *via* inflammatory cell chemotaxis, cytokine and chemokine release, and upregulation of vascular endothelial growth factor (VEGF) and matrix metalloproteinase-9 (MMP-9) (4, 5, 26). The endothelial cell swelling and cell membrane breakdown caused by cytotoxic edema may also increase the permeability of the BBB (34). After BBB injury, both the filtration coefficients of oncotic conductivity and the hydraulic conductivity rise (26), the water and macromolecular substances can more easily pass through the cell membrane and enter the interstitial tissue of the brain, causing vasogenic edema. The intracranial pressure, blood pressure, and concentration of intravascular osmotically active molecules influence the relevant hydraulic pressure and oncotic pressure, thereby affecting the formation of PHE (26). BBB opening is associated with rapid activation of the complement cascade. Complement fragments (e.g., C3a, C5a, and others) amplify the inflammatory response in a positive feedback loop to disrupt the BBB (35), producing anaphylatoxins and membrane attack complexes that lyse erythrocytes and thus promote the formation of iron-induced PHE (36).

### First Stage of PHE Formation

In the first few hours after ICH onset, during which the coagulation cascade is activated, the blood clot retraction, cell death, and brain atrophy induced by destruction of the hematoma produces a relatively large perihematomal space, leading to a reduction in the perihematomal hydrostatic pressure (37). The serum protein that is extruded secondary to the blood clot retraction leads to an increase in the interstitial oncotic pressure (38). Together, these changes induce the initial transport of water into the brain tissue, leading to edema. An important point to note is that the ionic edema driven by the cytotoxic edema dominates the first stage of PHE. The development of cytotoxic edema reportedly involves aberrant regulation of ion transport channels expressed on vascular endothelial cells (e.g., Na-K-Cl cotransporter 1 (NKCC1), which plays a role in the brain edema associated with ischemic stroke) and the sulfonylurea receptor 1–transient receptor potential cation channel subfamily M member 4 (SUR1-TRPM4) (39).

In the early stage of ICH, after transition from the closed to open state of the NKCC1 secondary to perihematomal glutamate deposition (28), Na^+^ is transported across the membrane to the interstitial tissue; this transport is driven by the transendothelial forces produced by the cytotoxic edema. Ionic edema forms as the Cl^−^ and water follow the movement of Na^+^ to maintain electrical and osmotic neutrality (40). When adenosine triphosphate depletion occurs, the NKCC1 closes while the SUR1-TRPM4 opens, allowing the Na^+^ and water to be transferred to the interstitial tissue along their gradient, resulting in edema (39). Nevertheless, the associated molecular pathways of ionic edema remain poorly understood, and the involvement of cytotoxic edema in PHE is not well defined.

### Second Stage of PHE Formation

Within 2 days after ICH onset, the vasogenic edema induced by the inflammatory immune response dominates the second stage of PHE (4–6). Numerous molecules are involved in this response, and four main pathways have been recognized. First, the thrombin and mechanical destruction of the hematoma activate Toll-like receptor 4 and the nuclear factor κB (NF-κB) pathway. NF-κB activates and regulates the transcription of cytokines, chemokines, and MMPs, leading to BBB dysfunction (41). The expression of Toll-like receptor 4 begins at 6 h after ICH onset and persists for almost 7 days, also triggering microglial activation (42, 43). Second, the activated thrombin induces the expression of chemokines and adhesion molecules, promoting the recruitment and infiltration of inflammatory cells (e.g., neutrophils, macrophages, and lymphocytes) to perihematomal sites (44). These recruited inflammatory cells release cytokines, reactive oxygen species, tumor necrosis factor-α, and MMPs, leading to BBB injury (45). Chemotaxis of neutrophils and polymorphonuclear leukocytes begins shortly after ICH onset and peaks at 3 days (46). Third, thrombin can further activate astrocytes and microglia *via* proteinase-activated receptor 4. The hyperactivation of microglia may exaggerate neuroinflammation through the secretion of reactive oxygen species, tumor necrosis factor-α, and cytokines (4, 5). Because microglial activation peaks at 3 days and significantly decreases 1 week after ICH (47), the SBI induced by these activated microglia is still maintained despite the fact that the leukocyte infiltration gradually resolves after 2 to 3 days (4). Notably, M2 microglia promote endogenous clearance of the hematoma following ICH (48). However, in the acute and subacute phases, the M2-dominant microglia quickly switch to M1-phenotype microglia (49); these M1 microglia excessively release destructive proinflammatory mediators and neurotoxic substances, leading to BBB dysfunction, PHE, and neurologic dysfunction (50). Thus, conversion of M1 to M2 microglia may be a potential treatment modality for ICH-induced SBI. Fourth, the activated complement cascade increases the production of anaphylatoxins and chemokines, resulting in increased permeability of the BBB (35).

### Third Stage of PHE Formation

Although erythrocyte lysis is initiated within 24 h after ICH (4), erythrocyte lysis with resultant hemoglobin and iron-related toxicity still dominates the PHE process 3 days after ICH onset. Specifically, the erythrocytes are dissolved to hemoglobin by complement-produced membrane attack complexes, and the hemoglobin is then oxidized into methemoglobin, which rapidly liberates its heme. The heme is then degraded into free iron *via* heme oxygenase enzymes (36). An experimental rat model showed that iron deposition occurs within 24 h after ICH, peaks after 7 days, and is maintained at a high concentration for at least 2 weeks (51). The free iron also stimulates the production of reactive oxygen species and MMP-9, promoting an inflammatory reaction and BBB dysfunction (45). The deposition of hemosiderin upregulates AQP-4, which exacerbates the brain edema and peaks at 3 to 7 days (5, 52). The hemoglobin and heme can also directly activate Toll-like receptor 4, microglia, and the NF-κB pathway to further promote the inflammatory reaction (53, 54). Consequently, the third stage of PHE is also arguably a delayed stage of vasogenic edema induced by erythrocyte lysis.

## Measurement of PHE

During head MRI, PHE appears as a hyperintense lesion with a clear boundary on T2-weighted imaging and fluid-attenuated inversion recovery. Although these two imaging techniques are the best choices for measuring the volume of PHE, the use of MRI may not be possible in emergency settings. Additionally, measuring PHE by CT examination is challenging because the PHE may be difficult to distinguish from periventricular leukoaraiosis over time. Manual segmentation of PHE is undoubtedly reliable but is not practical because it is a highly laborious process. Moreover, the consistency of manual segmentation may be lower than that of automatic segmentation. A CT value-based semiautomatic segmentation tool has been applied in numerous studies of PHE (10, 21, 55). The use of this segmentation tool requires researchers to manually delineate the region of interest, after which all voxels within the threshold range of edema (5–33 HU) are accumulated to obtain the PHE volume. Volbers et al. (56) verified that the performance of semiautomatic PHE segmentation is more consistent than that of manual PHE segmentation and shows less interference by periventricular leukoaraiosis. Urday et al. (57) found that the PHE volume obtained from both semiautomatic segmentation and manual segmentation was similar (*R* ^2^ = 0.98, *p* < 0.0001). However, there are potential limitations regarding the accuracy of semiautomatic segmentation because there may be a certain degree of variability of neuroanatomic characteristics among individual patients. External validation of semiautomatic segmentation is required to verify its efficacy in real clinical settings, and this method still needs to be refined to reduce the processing time of generating segmentation. Deep learning methods based on convolutional neural networks have become another option for automatic PHE segmentation. Zhao et al. (58) developed a deep learning model based on an U-Net for PHE segmentation. However, the best dice value was only 0.71. These findings indicate that automatic PHE segmentation is considerably more difficult than hematoma segmentation because of the lower clarity of PHE on CT scans (58, 59), necessitating refinement of the performance of automatic PHE segmentation.

Various parameters reflecting the severity of PHE have been adopted by separate studies (9, 10, 60). An indicator of the absolute PHE volume or absolute change (or absolute growth rate) of the PHE volume is frequently used to assess PHE and its progression. However, it would be inappropriate to evaluate the true effect of PHE on patients’ prognosis by using these indicators because the absolute PHE volume is strongly dependent on the initial ICH volume (61, 62). The relative PHE volume is the ratio of the absolute PHE volume to the ICH volume, which enables researchers to better compare the severity of PHE in patients with different initial ICH volumes. However, when using the relative PHE volume or the relative change of the PHE volume to predict the prognosis in patients with ICH, mismatch between the predictive results and the actual outcome may be obtained when the initial hematoma is small (63). Furthermore, it would be inappropriate to evaluate the severity of edema in patients with a hematoma that has ruptured into the ventricles using the indicator of the relative PHE volume because erythrocyte lysis in the brain parenchyma is alleviated by the dilutional effects of the cerebrospinal fluid. The absolute or relative peak PHE volume has also been used as an indicator of PHE severity and was considered to be associated with the 3-month neurological outcome (20). However, it is difficult to obtain an accurate peak PHE volume in the clinical setting. No effective methods with which to predict the peak PHE growth rate and volume in individual patients have yet been established. The EED represents the average thickness in centimeters of the edema beyond the boundary of the hematoma (64). Wu et al. (10) found that an unexpected EED within 72 h of onset was associated with a 6-month mortality rate. The EED is calculated using the following formula: $\sqrt[{3}]{\frac{\text{PHE Volume}+\text{ICH Volume}}{4/3\pi }}-\sqrt[{3}]{\frac{\text{ICH Volume}}{4/3\pi }}$ . However, the EED is calculated based on the assumption that both the hematoma and the total lesion (hematoma + PHE) are ellipsoid, introducing controversy into use of the EED to evaluate the PHE severity with irregularly shaped ICH (60).

## Risk Factors for PHE

### Imaging Features

Numerous studies have demonstrated that the initial hematoma volume determines PHE formation (9, 10, 12, 65), which shows good agreement with the aforementioned mechanism in which a greater ICH volume is associated with stronger thrombin cascades, erythrocytes lysis, and ICH-related toxicity. Several studies have revealed that a higher percentage of surgical hematoma removal results in slower PHE growth (24, 66). However, Sprügel et al. (20) indicated that the surface of the hematoma, not the initial hematoma volume, is the primary driver of PHE growth because a smaller hematoma has a larger relative surface area, contributing to a higher PHE volume per unit of the hematoma’s surface area. The evidence that irregular ICH and relatively minor ICH (<30 ml) generate a higher relative PHE volume was verified, supporting the surface-driven hypothesis. Notably, however, irregular and relatively minor ICH has a higher relative, not absolute, PHE volume. A recent study showed that certain CT imaging signs, such as the blend sign, black hole sign, and island sign, are capable of predicting hematoma expansion (67) and are associated with PHE growth in the acute phase of ICH (68). However, there is currently no evidence supporting an association between hematoma expansion and PHE formation. Rodriguez-Luna et al. (12) found that patients with spot signs on baseline CTA had a larger absolute PHE volume. Nevertheless, using the absolute PHE to predict hematoma expansion would be inappropriate because the PHE strongly depends on the initial hematoma volume. There is great controversy regarding the severity of PHE in different ICH locations. Sprügel et al. (20) found that lobar ICH had a larger initial PHE volume and higher early PHE growth rate. However, there was no significant difference in the peak PHE volume between deep and lobar ICH after adjusting for the hematoma volume (20). McCarron et al. (69) found that PHE was not affected by the location of ICH within 24 h after onset. In contrast, Grunwald et al. (11) found that the growth rate of lobar PHE within 24 h of ICH was significantly higher than that of deep ICH and was associated with the 90-day mortality rate. However, there was no significant difference in the growth rate of PHE within 72 h of onset between lobar and deep ICH. Cerebral amyloidosis was found to be a common cause of lobar ICH, which has localized anticoagulant and thrombolytic properties (70). However, we found no evidence indicating that lobar PHE is significantly smaller than deep PHE. We speculate that different shapes and growth patterns of PHE exist in different locations, and these differences are probably due to the different morphologies of the hematoma and the heterogeneity of the targeted population with diverse characteristics of ICH.

### Baseline Characteristics

In addition to imaging features, the patient’s baseline neurological status (e.g., as measured by the National Institutes of Health Stroke Scale score, Glasgow Coma Scale score, and other indexes) is also significantly associated with PHE progression (10). Advanced age is an independent risk factor for PHE (10, 61). However, Peng et al. (21) indicated that younger patients are more likely to develop delayed PHE formation, which may be due to age-related differences in brain atrophy. Whether sex influences PHE continues to be debated. Wagner et al. (71) found that the PHE volume in women with supratentorial ICH is lower than that in men which may be associated with the higher levels of estrogen in women, enabling alleviation of iron-induced PHE. However, other studies have produced different or even contrary conclusions (16, 65). Because poorly controlled hypertension and unstable blood pressure at admission are assumed to be risk factors for hematoma expansion (72, 73), the role of blood pressure in PHE is also attracting interest. The Intensive Blood Pressure Reduction in Acute Cerebral Haemorrhage Trial (INTERACT) showed that a history of hypertension was positively correlated with the relative growth of PHE, whereas lower systolic blood pressure at admission was positively associated with the absolute growth of PHE (65). In the ICH Acutely Decreasing Arterial Pressure Trial (ICH ADAPT) performed by McCourt et al. (74), aggressive antihypertensive treatment (diastolic blood pressure of <150 mmHg) was found to affect neither the perihematomal cerebral blood flow nor PHE progression. A clinical trial of hypothermia administration for PHE revealed that a higher number of hypertension attacks at admission was associated with a larger initial PHE volume (61). Moreover, the Antihypertensive Treatment of Acute Cerebral Hemorrhage-2 (ATACH-2) trial also demonstrated that intensive antihypertensive therapy (target systolic blood pressure of 110–139 mmHg within 2 h) effectively reduced the relative expansion rate of PHE within 24 h after onset (62). In general, intensive antihypertensive therapy has been demonstrated to be safe in the treatment of PHE. However, whether the control of blood pressure mediates the PHE by modulating hematoma expansion is unclear. Previous studies have shown that a shorter interval between onset and the initial CT scan is associated with a higher risk of hematoma expansion and more rapid relative PHE growth (65, 75). However, Rodriguez-Luna et al. (12) indicated that the ICH onset time did not affect the relative PHE volume within 6 h of onset. Because obtaining a precise onset time is significant for determining the optimal treatment of ICH, whether the onset time affects the PHE needs to be further investigated. Additionally, the imaging features of ICH and PHE may help to predict the onset time.

### Laboratory Testing

Several laboratory parameters that have been confirmed to affect hematoma expansion, such as hyperglycemia, a high MMP-9 level, and a high white blood cell count, may also be positively associated with PHE progression (10, 61, 76, 77). Gusdon et al. (78) found that the ratio of neutrophils to lymphocytes is effective in predicting PHE growth. Because MMP-3, MMP-9, VEGF, and angiopoietin-1 are all related to vascular function, they might be predictive of vasogenic edema (79). A high RBC count and high hematocrit at admission are associated with delayed peak PHE (16). This may be relevant because a high RBC count and high hematocrit are indicative of higher RBC degradation, which has been identified as an essential factor for promoting PHE. A high platelet count promotes increases in the VEGF level and capillary permeability, thereby exacerbating PHE (16). A prolonged partial thromboplastin time is significantly associated with PHE growth (16, 61, 80). This could be due to a consumptive coagulopathy resulting from significant release of coagulation factors after ICH, manifesting as platelet dysfunction and prolonging the partial thromboplastin time (80, 81). Some studies have shown that hyperglycemia is associated with earlier PHE progression (10, 82), which might be due to the fact that hyperglycemia promotes an oxidative stress response with consequent BBB dysfunction (83). However, hyperglycemia can merely be a stress response to ICH instead of a contributing factor for PHE. Feng et al. (84) found that hyperglycemia did not significantly affect PHE after adjusting for the initial ICH volume.

### Others

Apolipoprotein E (Apo­E) has been considered an independent risk factor for lobar ICH (85). Apo-E plays an essential role in maintaining normal lipid homeostasis in the central nervous system (86) and mitochondrial resistance to oxidative stress (87). James et al. (88) found that *APOE-ϵ4* positivity was associated with a larger PHE volume. However, McCarron et al. (89) demonstrated that *APOE-ϵ4* positivity was not associated with PHE after adjusting for race, age, and type of bleeding. These two studies reached different conclusions, which might be related to the selection of different time windows for PHE observation. Further studies are still needed to understand the role of Apo­E in PHE progression.

## Impact of PHE on ICH Prognosis

Whether PHE directly affects the prognosis of ICH remains controversial. From a pathogenesis perspective, PHE formation in the ultra-early stage of ICH might be clinically meaningful because the blood clot retraction and activated thrombin cascade are effective in promoting hemostasis (90). A recent magnetic resonance spectroscopy study of an experimental ICH model showed that the recovery of *N*-acetylaspartate, choline, and creatine was faster in the PHE area than in the non-PHE area, suggesting that the PHE may maintain the integrity of the perihematomal tissue and provide a protective buffer against irreversible impairment (91). Levine et al. (92) reported that a higher absolute PHE volume was associated with lower 90-day mortality. Similar results were obtained by two other studies using the indicator of the relative PHE volume to predict the functional status after ICH (90, 93). However, real-world cohort studies, such as the INTERACT-1/2 trials, have shown that absolute PHE growth is associated with poor outcomes of ICH (94). The absolute or relative PHE growth rate within 72 h of onset is considered an independent predictor of death and poor functional prognosis (modified Rankin scale score of ≥3) (9, 11, 65). Urday et al. (60) found that the PHE expansion rate within 24 h significantly affected mortality within 90 days of ICH onset even after adjusting for the initial hematoma volume. Wu et al. (10) found that patients with a larger initial EED were more likely to develop a significant midline shift and brain herniation, which were related to the 6-month mortality rate after ICH. However, not all studies have shown that PHE significantly affects the ICH prognosis after adjusting for the initial hematoma volume (61, 65). Appelboom et al. (95) found that the absolute PHE volume is correlated with the prognosis of patients with an ICH volume of <30 ml, whereas the relative PHE volume does not affect the modified Rankin scale score upon hospital discharge. The impact of PHE from different ICH locations on the prognosis remains controversial. The finding that lobar PHE was not associated with the 3-month modified Rankin scale score may have resulted from different morphological characteristics of hematomas among different ICH locations (11). The ATACH-2 trial showed that PHE of basal ganglia hemorrhage, but not thalamic hemorrhage, was associated with the 3-month prognosis. This result was most likely obtained because thalamic hemorrhage is more possible to develop intraventricular hemorrhage, which is a potential confounder (62). Peng et al. (21) reported that delayed PHE formation was an independent predictor of a poor prognosis at discharge. In their study, delayed PHE was defined as an absolute PHE volume that is 3 ml greater at 12 to 20 days than at 5 to 9 days. This is a particularly interesting finding because it seems revealed that a mild unsteady state of PHE on the delayed phase instead of acute phase significantly affects the prognosis. Notably, however, there is currently no consensus on the definition of delayed PHE, and whether delayed PHE affects the prognosis of ICH remains unclear.

Because the time of the follow-up CT scan differed among the population of various retrospective studies on PHE, and because heterogenous metrics reflecting the severity of PHE were adopted by separate studies, the ability of PHE to predict prognosis remains unclear. The results of PHE-based prognostic studies are summarized in **Tables 1**–**4**.

**Table 1:** Summary of prognostic research of PHE—the PHE was associated with poor ICH outcome.

Study	Method	Patient	Focused Time	Imaging Method	PHE Measurement	PHE Metric	Prognostic Marker
Peng et al. (21)	Retrospective, single-center	*N* = 121, supratentorial ICH	5–9, 12–20 days after onset	CT	Semiautomated calculation based on CT Hounsfield units	Delayed perihematomal edema: the volume of absolute PHE in 12–20 days is 3 ml larger than that in 5–9 days	Discharge mRS score 2–6
Chen et al. (55)	Retrospective, single-center	*N* = 138, ICH	24 h after onset	Baseline: CTP; follow-up: CT	Semiautomated calculation based on CT Hounsfield units	Absolute/relative PHE volume	3-month mRS score 3–6
Wu et al. (10)	Retrospective, single-center	*N* = 861, supratentorial/cerebellar ICH	3 weeks after onset	CT	Semiautomated calculation based on CT Hounsfield units	Edema extension distance	6-month mortality; brain herniation
Murthy et al. (9)	Retrospective, Virtual International Stroke Trials Archive	*N* = 596, ICH	72 h after onset	CT	Semiautomated calculation based on CT Hounsfield units	Expansion rates	3-month mRS score 3–6
Urday et al. (60)	Retrospective, single-center	*N* = 139, supratentorial ICH	24 and 72 h after onset	CT	Automatic segmentation	Expansion rates; absolute/relative PHE volume	3-month mortality; 3-month mRS score 3–6
Volbers et al. (18)	Retrospective, single-center	*N* = 220, supratentorial ICH	12 days after onset	CT	Semiautomated calculation based on CT Hounsfield units	Expansion rates; peak absolute-PHE volume; peak relative-PHE volume	Discharge mRS score 4–6
Yang et al. (94)	Retrospective, INTERACT1/INTERACT2	*N* = 1,138, ICH	24 h after onset	CT	Semiautomated calculation based on CT Hounsfield units	Expansion rates	3-month mortality; 3-month mRS score 3–6
Murthy et al. (63)	Retrospective, Virtual International Stroke Trials Archive	*N* = 596, ICH	72 h after onset	CT	Semiautomated calculation based on CT Hounsfield units	Expansion rates	3-month mRS score 3–6
Staykov et al. (19)	Retrospective, single-center	*N* = 219, supratentorial ICH	Days 1–21; day ≥22	CT	Semiautomated calculation based on CT Hounsfield units	Absolute/relative PHE volume	In-hospital mortality
Inaji et al. (17)	Retrospective, single-center	*N* = 14, ICH	1, 3, 7, 14, and 28 days after onset	CT	Unclear	Absolute PHE volume	In-hospital NIHSS score

ICH, intracerebral hemorrhage; CT, computed tomography; PHE, perihematomal edema; mRS, modified Rankin Scale; CTP, CT perfusion; INTERACT, Intensive Blood Pressure Reduction in Acute Cerebral Hemorrhage Trial.

**Table 2:** Summary of prognostic research of PHE—the PHE was associated with improved ICH outcome.

Study	Method	Patient	Focused Time	Imaging Method	PHE Measurement	PHE Metric	Prognostic Marker
Gupta et al. (93)	Prospective, single-center	*N* = 44, supratentorial ICH	24 to 72 h after onset	CT	Manual segmentation	Relative PHE volume	3-month mRS score 0–2
Levine et al. (92)	Retrospective, single-center	*N* = 98, warfarin-related ICH and noncoagulopathic ICH	24 h after onset	CT	Manual segmentation	Absolute PHE volume	3-month mortality
Gebel et al. (90)	Prospective, single-center	*N* = 142, ICH	3 and 20 h after baseline image	CT	Semiautomated calculation based on CT Hounsfield units	Baseline relative PHE volume	3-month mRS score 0–2

ICH, intracerebral hemorrhage; CT, computed tomography; PHE, perihematomal edema; mRS, modified Rankin Scale.

**Table 3:** Summary of prognostic research of PHE—the PHE was not associated with ICH outcome.

Study	Method	Patient	Focused Time	Imaging Method	PHE Measurement	PHE Metric	Prognostic Marker
Hervella et al. (61)	Retrospective, single-center	*N* = 795, ICH	7 days after onset	CT	ABC/2 method; automatic segmentation	Absolute PHE volume	3-month mRS score
Rodriguez-Luna et al. (12)	Prospective, multicenter	*N* = 353, ICH	24 h after onset	Baseline: CTA; follow-up: CT	Semiautomated calculation based on CT Hounsfield units	Absolute/relative PHE volume	Hematoma expansion
Qureshi et al. (96)	Retrospective, multicenter	*N* = 60, ICH	24 h after onset	CT	Semiautomated segmentation	Expansion rates	3-month mRS score 3–6
Arima et al. (65)	Retrospective, INTERACT	*N* = 296, ICH	24 and 72 h after onset	CT	Semiautomated calculation based on CT Hounsfield units	Expansion rates	3-month mRS score 3–6
Leira et al. (97)	Prospective, multicenter	*N* = 266, supratentorial ICH	48 h after onset	CT	ABC/2 method	Absolute/relative PHE volume	Early neurologic deterioration: CSS score decreased > or =1 point between admission and 48 h

ICH, intracerebral hemorrhage; CT, computed tomography; CTA, CT angiography; PHE, perihematomal edema; mRS, modified Rankin Scale; CSS, Canadian Stroke Scale; INTERACT, Intensive Blood Pressure Reduction in Acute Cerebral Hemorrhage Trial.

**Table 4:** Summary of prognostic research of PHE—the conflicting findings.

Study	Method	Patient	Focused Time/Imaging Method	PHE Measurement	PHE Metric	Prognostic Marker	Conflicting Findings
Leasure et al. (62)	Retrospective, the ATACH-2 randomized trial	*N* = 870, deep-supratentorial ICH	24 h after onset; CT	Manual segmentation	Expansion rates	3-month mRS score 4–6	Positive: basal ganglia PHE; negative: thalamus PHE
Grunwald et al. (11)	Retrospective, single-center	*N* = 115, supratentorial ICH	24 and 72 h after onset; CT	Automatic segmentation	Expansion rates	3-month mortality; 3-month mRS score 3–6	Positive: (1) 24 h deep/lobar PHE; (2) 72 h deep PHE; negative: 72 h lobar PHE
Lord et al. (98)	Retrospective, Virtual International Stroke Trials Archive	*N* = 376, ICH	24 and 72 h after onset; CT	Unclear	Absolute PHE volume	In-hospital neurological deterioration: a ≥2-point decrease in GCS or a ≥4-point increase in the NIHSS score	Positive: 0–24 h neurological deterioration; negative: 1–3 days neurological deterioration
Li et al. (99)	Prospective, single-center	*N* = 21, ICH	1, 3, and 7 days after onset; MRI	Manual segmentation	Absolute PHE volume; presence of cytotoxic edema	3-month mRS score 4–6	Positive: 72 h PHE; negative: (1) baseline PHE and (2) cytotoxic edema
Appelboom et al. (95)	Retrospective, single-center	*N* = 133, ICH	24 h after onset; CT	Semiautomated segmentation	Absolute/relative PHE volume	Discharge mRS score 3–6	Positive: absolute PHE volume; negative: relative PHE volume
Venkatasubramanian et al. (16)	Prospective, single-center	*N* = 27, ICH	21 days after onset; MRI	Manual segmentation	Expansion rates	In-hospital NIHSS; 3-month mRS score; 3-month eGOS	Positive: 48 h NIHSS; negative: 3-month mRS/eGOS score
Gebel et al. (90)	Prospective, single-center	*N* = 142, ICH	3 and 20 h after baseline image; CT	Semiautomated calculation based on CT Hounsfield units	Absolute/relative PHE volume	3-month mRS score 3–6	Positive: baseline relative PHE volume; negative: absolute PHE volume

Positive indicates the PHE was associated with poor/improved ICH outcome; negative indicates the PHE was not associated with ICH outcome.

ICH, intracerebral hemorrhage; CT, computed tomography; PHE, perihematomal edema; mRS, modified Rankin Scale; NIHSS, National Institutes of Health Stroke Scale; eGOS, extended Glasgow Outcome scale.

## Potential Therapies for PHE

### Targeted Strategies for Cytotoxic Edema

The formation of the perihematomal osmotic gradient, which is driven by cytotoxic edema, dominates PHE in the ultra-early phase of ICH (25, 38). Compared with mannitol, the currently available dehydrating drug that is commonly used to decrease the intracranial pressure, continuous infusion of hypertonic saline has been identified as a safe method for controlling PHE progression in the early phase of ICH, and it does not seem to affect the BBB (100, 101). Given the putative advantages of hypertonic saline in improving cerebral perfusion, Cook et al. (102) reported that hypertonic saline possesses a better capacity for controlling PHE and the corresponding intracranial hypertension than mannitol. However, in a recent multicenter randomized controlled trial, Roquilly et al. (103) found that continuous infusion of 20% hypertonic saline solution did not improve the neurological outcome 6 months after onset among patients with moderate to severe traumatic brain injury. As previously described, during the stage of cytotoxic edema, the SUR1-TRPM4 channel was confirmed to be upregulated, promoting ionic edema (5, 104). Jiang et al. (105) established a model of autologous blood-induced ICH and found that glibenclamide (a SUR1 inhibitor) effectively reduced the PHE volume, which was associated with cognitive deficit improvement. However, another study involving a model of collagenase-induced ICH showed that glibenclamide neither aggravated nor ameliorated the PHE volume or neurological dysfunction (106). Sheth et al. (107) conducted a double-blind, randomized controlled trial of patients with cerebral hemispheric infarcts and found that glibenclamide therapy significantly reduced the midline shift and MMP-9 level compared with the control group, revealing the potential role of glibenclamide in alleviating PHE after stroke. Additional clinical trials are needed to investigate preclinical strategies for cytotoxic edema.

### Targeted Strategies for Vasogenic Edema

The thrombin cascades, inflammatory response, and BBB dysfunction have been confirmed to exert essential functions in PHE formation (4). Thus, treatments targeting critical molecules in the formation of vasogenic edema (such as VEGF, MMPs, and AQPs) may be promising (5, 108). In one study, the anti-inflammatory drug fingolimod alleviated the progression of PHE and improved the functional independence of patients with ICH at 90 days (109). However, patients with a sizeable initial hematoma (>30 ml) were not included. Given that PHE is strongly dependent on the primary hematoma, whether fingolimod can benefit critically ill patients with ICH remains to be explored. Statins are HMG-CoA reductase inhibitors that exert their neuroprotective effects by anti-inflammatory actions and facilitation of neo-angiogenesis (110). Statins have also been found to reduce the absolute or relative PHE volume (111), and most relevant studies have shown that statin use does not increase the risk of ICH recurrence (112). However, the effect of statins on the growth rate of PHE has not been demonstrated. Celecoxib is a selective cyclo-oxygenase 2 receptor inhibitor that attenuates the inflammatory reaction and edema by inhibiting the generation of prostaglandins (113). A multicenter randomized controlled trial confirmed the efficacy of using celecoxib to reduce the expansion rate of PHE (114). However, because the time to initial CT was longer in the celecoxib group than in the control group in that study, the primary outcome was defined as a ≥20% change in PHE from onset to an average of 1 week, which may be inappropriate because a longer time to initial CT may represent a steady state for PHE (10, 98). Antiadrenergic drugs such as β-blockers and α2-agonists have also been used to manage hypertension in patients with ICH. A retrospective analysis of a prospective cohort of patients with cerebral hemorrhage (CHANT trial) showed that the administration of antiadrenergic drugs effectively reduced PHE within 72 h after onset (115), suggesting that a reduction of central/peripheral sympathetic activity attenuates neuroinflammation and thereby alleviates the PHE. Notably, the reduction of PHE might not have been due to the antihypertensive actions of these antiadrenergic drugs because other kinds of blood pressure-lowering drugs did not result in the same degree of PHE reduction. The transcription factor peroxisome proliferator-activated receptor gamma (PPAR-γ) plays a significant role in modulating the biomarkers of oxidative stress and inflammation (116). One study showed that the PPAR-γ agonist rosiglitazone significantly reduced the expression of proinflammatory genes such as tumor necrosis factor-α, interleukin-β, and MMP-9 in a rat model of ICH and consequently attenuated the SBI (117). However, there is a scarcity of clinical trial data regarding the use of PPAR-γ agonists in patients with ICH. The iron chelator deferoxamine is a potential candidate for ICH treatment, and its effectiveness in alleviating PHE has been confirmed in experimental models of ICH (118, 119). A meta-analysis of the efficacy of deferoxamine in an experimental ICH model showed that deferoxamine reduced the brain water content by 85.7%, although the effect lasted for only 24 h after onset (120). However, it is discouraging to note that a double-blind, randomized controlled clinical trial showed no association between administration of deferoxamine mesylate and better neurological outcomes in patients with ICH (121). Indeed, because deferoxamine is characterized by a small effect size, it would be better to enroll a considerably high number of patients to verify the drug’s utility and validity when conducting studies targeting a small effect size. Li et al. recently performed an unbiased genome-wide transcript sequencing study for surgical removal of perihematomal brain tissue in patients with ICH and identified abundant expression of formyl peptide receptor 1 (FPR1), which promotes neuroinflammatory reactions. Under the screening of a computer-aided drug design system, the research group further selected an FPR1 inhibitor (T-0080) that can cross the BBB and successfully reduced the PHE by about 35% in experimental ICH models to improve the neurological status (122). This FPR1 inhibitor may be a promising candidate for ICH therapy. Antidiuretic hormone maintains the brain water content by regulating the permeability of capillaries. Conivaptan is an antidiuretic hormone receptor antagonist that was confirmed to reduce brain edema and repair the BBB function in an experimental ICH model (123). The effects of conivaptan on the treatment of PHE might be correlated with a reduction in the expression of AQP-4 (124). A recent phase I clinical trial verified the safety of conivaptan in the treatment of PHE (125). A phase II clinical trial of antidiuretic hormone receptor antagonists is urgently needed to further explore their efficacy on SBI in patients with ICH.

### Comprehensive Treatments

The MISTIE II trial showed that minimally invasive surgery combined with tissue-type plasminogen activator effectively reduces the PHE volume (126). In the MISTIE III trial, the patients in the surgery group had improved neurological outcomes at 1 year when no more than 15 ml of hematoma remained at the end of the treatment (127). Similarly, a recent study showed that minimally invasive endoscopic surgery for ICH evacuation alleviated the postoperative PHE progression. A higher percentage of hematoma removal results in slower PHE growth (24). Although hematoma evacuation surgery has been shown to be a promising treatment for PHE, whether the patient’s prognosis can be significantly improved remains unclear. Fung et al. (23) reported that patients who underwent simple decompression surgery (without hematoma removal) developed more severe PHE than patients in the control group. However, there was no significant difference in the 90-day neurological function between the decompressive craniectomy group and the control group (23). Perhaps when the surgical indications for ICH become more detailed and standardized, the benefits of surgical interventions in reducing the complications of SBI (e.g., PHE) will gradually emerge.

Supportive treatments, including hypothermia therapy (61, 128), intensive antihypertensive therapy (62), and hyperbaric oxygen therapy (129), have also been reported to reduce PHE growth. However, the number of existing clinical studies is small, and inevitable bias and confounding factors have limited these studies. A large‐scale prospective follow‐up study for validation is warranted.

## Conclusion

Because of the lack of significant progress in treating hematomas in patients with ICH, damage secondary to ICH (especially PHE) has recently become a promising therapeutic target. This article has reviewed the mechanisms and growth patterns of PHE after ICH and has introduced potential treatments. However, previous studies have various limitations. For example, the sample sizes, especially in studies involving brain MRI, are limited; no standard indicator of PHE severity has been established; and prospective primary data are entirely lacking. Randomized controlled trials of PHE are urgently needed. Furthermore, researchers are expected to employ more effective measuring technologies to analyze high-quality imaging data, effectively explore the laws involved in PHE growth, and develop new therapeutic options for patients with ICH.

## Author Contributions

All authors listed have made a substantial, direct, and intellectual contribution to the work and approved it for publication. All authors contributed to the article and approved the submitted version.

## Funding

This work was supported by the National Natural Science Foundation of China (82001389), Chinese Academy of Medical Sciences (CAMS) Innovation Fund for Medical Science (2020-I2M-C&T-B-031), CAMS/PUMC Postgraduate Teaching Innovation Fund (No. 10023201900107), National Key R&D Program of China (2018YFA0108600), and Huazhong University of Science and Technology Union Shenzhen Hospital Fund (NS202001).

## Conflict of Interest

The authors declare that the research was conducted in the absence of any commercial or financial relationships that could be construed as a potential conflict of interest.

## Publisher’s Note

All claims expressed in this article are solely those of the authors and do not necessarily represent those of their affiliated organizations, or those of the publisher, the editors and the reviewers. Any product that may be evaluated in this article, or claim that may be made by its manufacturer, is not guaranteed or endorsed by the publisher.

## Abbreviations

Apo­E, apolipoprotein E; AQPs, aquaporin; ATACH, antihypertensive treatment of acute cerebral hemorrhage; ATP, adenosine triphosphate; BBB, blood–brain barrier; CT, computed tomography; CTA, CT angiography; DC, decompressive craniectomy; EED, edema extension distance; FPR1, formyl peptide receptor 1; ICH ADAPT, ICH Acutely Decreasing Arterial Pressure Trial; ICH, intracerebral hemorrhage; INTERACT, Intensive Blood Pressure Reduction in Acute Cerebral Haemorrhage Trial; MISTIE, minimally invasive surgery and rt-PA in ICH evacuation; MRI, magnetic resonance imaging; MMP, matrix metalloproteinases; NKCC1, Na–K–Cl cotransporter 1; NF-κB, nuclear factor κB; PHE, perihematomal edema; PT, partial thromboplastin; PPAR-γ, peroxisome proliferator activated receptor gamma; RBC, red blood cell; STICH, Surgical Trial in Intracerebral Hemorrhage; SUR, sulfonylurea receptor; SUR1–TRPM4, sulfonylurea receptor 1–transient receptor potential cation channel subfamily M member 4; TPA, tissue-type plasminogen activator; VEGF, vascular endothelial growth factor.
